# Emergence of Poultry-Associated Human *Salmonella enterica* Serovar Abortusovis Infections, New South Wales, Australia

**DOI:** 10.3201/eid3004.230958

**Published:** 2024-04

**Authors:** Michael Payne, Sarah Williamson, Qinning Wang, Xiaomei Zhang, Vitali Sintchenko, Anthony Pavic, Ruiting Lan

**Affiliations:** University of New South Wales, Sydney, New South Wales, Australia (M. Payne, X. Zhang, R. Lan);; Birling Laboratories, Bringelly, New South Wales, Australia (S. Williamson, A. Pavic);; Institute of Clinical Pathology and Medical Research–NSW Health Pathology, Westmead Hospital, Westmead, New South Wales, Australia (Q. Wang, V. Sintchenko);; University of Sydney, Sydney (V. Sintchenko)

**Keywords:** Salmonella enterica Abortusovis, bacteria, food safety, enteric infections, zoonoses, genomic epidemiology, comparative genomics, human infection, poultry, New South Wales, Australia

## Abstract

*Salmonella enterica* serovar Abortusovis is a ovine-adapted pathogen that causes spontaneous abortion. *Salmonella* Abortusovis was reported in poultry in 2009 and has since been reported in human infections in New South Wales, Australia. Phylogenomic analysis revealed a clade of 51 closely related isolates from Australia originating in 2004. That clade was genetically distinct from ovine-associated isolates. The clade was widespread in New South Wales poultry production facilities but was only responsible for sporadic human infections. Some known virulence factors associated with human infections were only found in the poultry-associated clade, some of which were acquired through prophages and plasmids. Furthermore, the ovine-associated clade showed signs of genome decay, but the poultry-associated clade did not. Those genomic changes most likely led to differences in host range and disease type. Surveillance using the newly identified genetic markers will be vital for tracking *Salmonella* Abortusovis transmission in animals and to humans and preventing future outbreaks.

*Salmonella enterica* serovar Abortusovis is a host-restricted pathogen of sheep that causes invasive disease and spontaneous abortion (*1*–*3*). Transmission is thought to be limited to sheep; however, early studies identified other potential carriers (*4*). Because *Salmonella* Abortusovis is relatively rare, only a handful of studies have examined the molecular underpinning of its virulence. Other studies identified *astA*, *sodC1*, and *sseI* genes as virulence factors for invasive disease in lambs (*5*), and a mouse systemic infection model identified the *spv* toxin encoded on a plasmid as essential for virulence (*6*,*7*).

Previous examination of *Salmonella* Abortusovis epidemiology has exclusively relied on nongenomic molecular methods (*8*–*11*). Pulse-field gel electrophoresis (PFGE) and insertion sequence 200 fingerprinting have been used to identify clonal lineages in geographically distinct regions in Italy, Spain, Iran, and Switzerland (*3*,*10*–*13*). Cases and outbreaks in sheep have been described in several countries including Spain, Croatia, Switzerland, and Italy (*8*–*10*). However, the incidence of *Salmonella* Abortusovis animal infections in those and other countries is estimated to be underreported (*9*).

*Salmonella* Abortusovis is a reportable disease in sheep in Australia. Before 2009, the bacterium had not been reported in any animal (*14*). However, in 2009, *Salmonella* Abortusovis was detected in commercial poultry flocks in Australia (*15*) and was the second most common serovar in meat chickens in the country in 2016 (*16*). We examined the sudden emergence and proliferation of this serovar in poultry and humans in New South Wales (NSW), Australia, to investigate its evolution and implications to public health.

## Methods

### Isolate Sources and Metadata 

**Figure 1:**
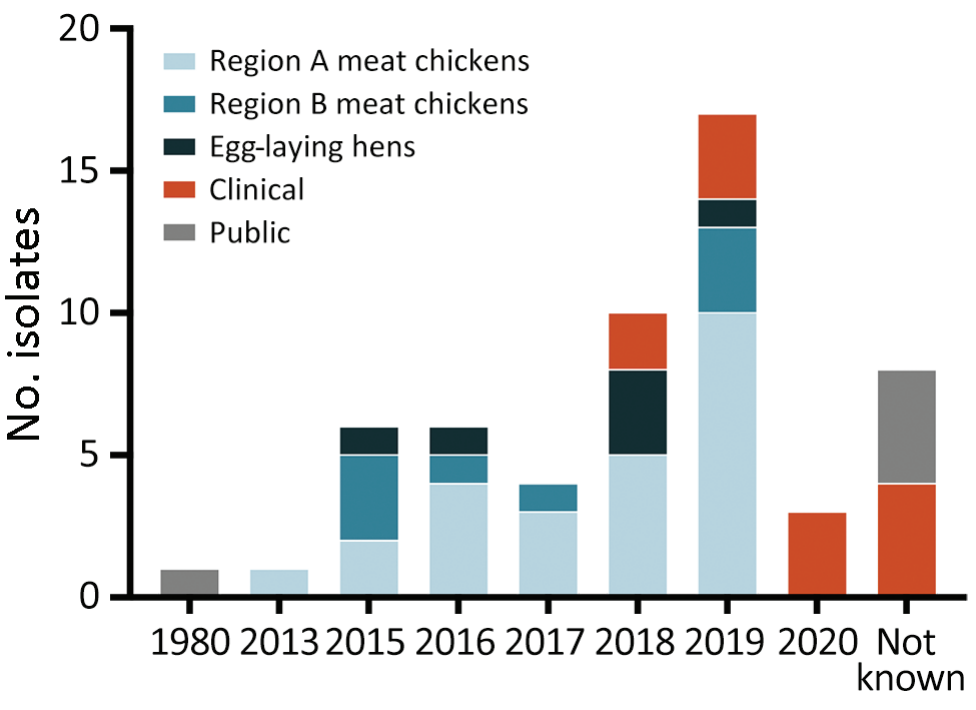
Temporal and geographic distribution of isolates in a study of the emergence of poultry-associated human *Salmonella enterica* serovar Abortusovis infections, New South Wales, Australia. Colors represent the region of isolation, isolates from human cases, and publicly available genomes.

We analyzed genomes and metadata of 56 *Salmonella* Abortusovis isolates (Table 1; Figure 1). Of those isolates, 51 were from Australia, 47 of which we sequenced in this study. Poultry isolates from Australia were collected during 2013­–2019 and were divided into region A meat chickens (25 isolates), region B meat chickens (8 isolates), and egg-laying hens (6 isolates). Of the 33 isolates from regions A and B, 4 were from feed ingredients, 20 were from 10 farms in region A, and 8 were from 7 farms in region B. Multiple isolates were sampled from 5 region A farms and 1 region B farm. Human clinical isolates were collected during 2018–2020.

**Table 1:** Characteristics of isolates used in a study of the emergence of poultry-associated human *Salmonella enterica* serovar Abortusovis infections, New South Wales, Australia*

Country	Bacterial source	Data source	No. isolates	Data type†
Australia	Poultry	This study	39	Illumina reads
Australia	Clinical	This study	8	Illumina reads
Australia	Clinical	NCBI	4	Assembly
Italy	Ovine	NCBI	1	Assembly
France	Unknown	NCBI	1	Illumina reads
Unknown	Unknown	NCBI	3	Illumina reads

*NCBI, National Center for Biotechnology Information (https://www.ncbi.nlm.nih.gov). †Illumina (https://www.illumina.com).

### Sequencing and Assembly

We assembled draft genomes for all 47 isolates sequenced in this study and assembled complete genomes for 4 isolates (Table 1; Appendix 1; Appendix 2 Table 1). We submitted all raw sequencing data to the National Center for Biotechnology Information BioProject database (no. PRJNA993380).

### Phylogenetic Analyses

We used the complete genome of strain 180121-R1 as the reference because it was the earliest representative of the poultry-associated clade isolate in Australia. We used Snippy version 4.6.0 (*17*) to call single-nucleotide polymorphisms (SNPs) for both assemblies and read sets and to generate core SNP alignments. We used the core SNP alignment as an input to custom Python scripts (https://github.com/mjohnpayne/Aus_Abortusovis) to perform pairwise distance analysis. Then we used IQ-TREE versions 2.0.3 (*18*) with default settings to generate a phylogeny for all 56 genomes. We generated a multilocus sequence typing (MLST)–based phylogeny from all sequence types (STs) within 5 alleles of ST768 and visualized all phylogenies by using iTOL (*19*) (Appendix 1).

### Bayesian Analysis

We used BEAST version 2.6.3 (*20*) and 10,000,000 Markov chain Monte Carlo chain length to perform Bayesian phylogenetic reconstruction. A general time-reversible site model with a strict clock and coalescent constant population had the best effective sample size across 3 replicates, and we used that model to provide estimates of the most recent common ancestor (MRCA) date and evolutionary rate (Appendix 1).

### Pan-Genome Analysis

We identified clade-specific genes by running roary version 3.13.0 (*21*) and default settings to define the pan-genome, and scoary version 1.6.16 (*22*) and default settings to identify clade-specific genes. We verified genes that were >80% sensitive and 100% specific for 1 clade by using KMA version 1.3.17 (*23*) and default settings to detect genes that were called absent because of assembly or annotation issues. We determined the presence of intact phages and plasmids in draft genomes by using KMA version 1.3.17 and default settings to search raw read data for phages and plasmids that were identified in complete assemblies.

### Pseudogene and Genome Size Analysis

We used pseudofinder version 1.1 and default settings and a database of all uniprot protein sequences from *Salmonella* strains to detect pseudogenes (*24*). To compare genome size between the 2 *Salmonella* Abortusovis clades and exclude plasmids, we used the draft genome of isolate 405580-R1 to represent the poultry-associated clade because it lacks plasmids in the complete genome assembly. Similarly, the draft genome of ERR230420 appears to lack the pSLT-like plasmid likely found in the ovine-associated clade. We used draft genomes because the ovine-associated clade does not contain a complete genome for comparison and repetitive elements are often not assembled using Illumina (https://www.illumina.com) data. An isolate or isolate DNA was not readily available to generate a complete genome for the ovine-associated clade for this study.

### Virulence Factor Identification

We searched all assemblies by using ABRicate version 1.0.1, the virulence factor database (VFDB), and plasmidfinder gene database by using mincov and minid set to 80 (*25**–**27*). We validated ABRicate results by using KMA version 1.3.17 for raw read mapping (Appendix 1). We used PHASTEST to identify prophages in complete genomes (*28*). We used the same datasets and approach used in a previous study to identify and select *Salmonella* Abortusovis–specific gene markers (*29*) (Appendix 1).

## Results

### Distinct Poultry-Associated Clade

**Figure 2:**
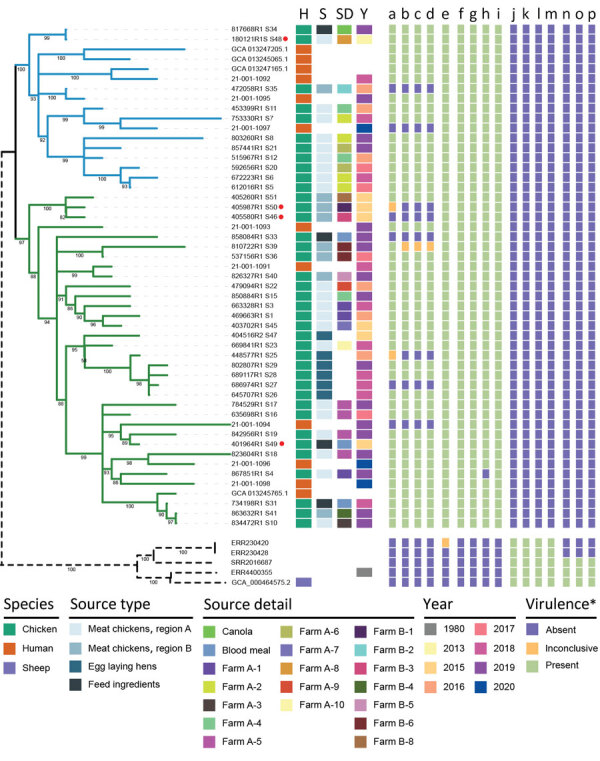
Phylogenetic relationships of isolates and associated metadata in a study of the emergence of poultry-associated human *Salmonella enterica* serovar Abortusovis infections, New South Wales, Australia. A maximum-likelihood phylogeny of all 56 isolates including subclade annotation and relevant metadata. Branches colors indicate subclades 1 (blue) and 2 (green) and bootstrap support values are indicated at tree nodes. Branch lengths for 5 international isolates were shortened for clarity (dashed lines). Red dots indicate complete genomes. Colors in columns indicate host species, source type, source details, year of isolation, and presence or absence of virulence genes. Virulence genes were predicted by using abricate (https://github.com/tseemann/abricate), KMA (*23*), and VFDB (*25*): a) pSAbAus; b) yersinia HPI (11 genes); c) *ivy*; d) *cvaA*; e) *cdtB*; f) *cfaB*; g) *stfF*; h) *hdeB*; i) *eutEJGHABCLKR*; j) *astA*; k) *sodCI*; l) *ompN*; m) *hcp1*; n) pSLT-like plasmid; o) *spv* toxin (3 genes); p) Fimbriae *klf/fae* (6 genes). *Some virulence genes or islands have the same presence and absence pattern in all genes; those data are collapsed into 1 column and the number of genes are indicated in parentheses in the list. Inconclusive gene presence was assigned when only KMA identified the gene and it had <20% of the normalized genome coverage. H, host species; S, source; SD, source details; Y, year.

Phylogenetic analysis of the 56 isolates included in the study demonstrated that the 47 poultry and human clinical isolates from Australia represent a distinct poultry-associated clade that is at least 22,785 SNPs distant from the 9 *Salmonella* Abortusovis isolates from elsewhere, which formed an ovine-associated clade (Figure 2). The maximum pairwise SNP distance between isolates from Australia was only 42. Within the poultry-associated clade, we detected 2 subclades, 1 (subclade 1) containing 17 isolates and 1 (subclade 2) containing 34 isolates. Both subclades contained poultry isolates from regions A and B and human clinical isolates. Isolates from layer chickens were limited to a single lineage within subclade 2 and were not closely related to any human clinical isolates.

We also collected detailed source information that enabled a thorough examination of isolates at the farm level (Appendix 2 Table 2). We identified *Salmonella* Abortusovis in 2 feed ingredients, subclade 1 in canola and subclade 2 in blood meal, and in breeder hens that were progenitors of broiler chickens, suggesting possible modes of transmission. Among 17 farms, 6 had >1 isolate collected, 5 region A farms and 1 region B farm. Of note, 1 farm, A-4, contained isolates from both subclades 1 and 2, suggesting multiple introduction events (Figure 2). By contrast, all other farms contained isolates from only 1 subclade, and some were closely related across multiple years (i.e., farm B-6) suggesting long-term colonization by a single strain.

**Figure 3:**
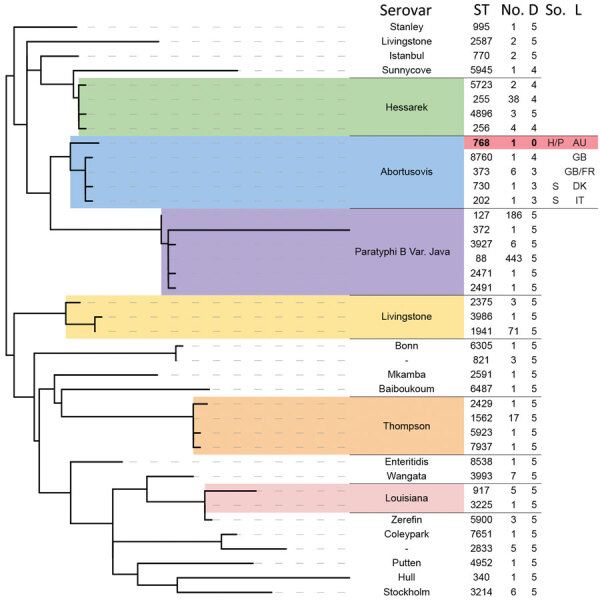
Phylogenetic relationship of other serovars to isolates in a study of the emergence of poultry-associated human *Salmonella enterica* serovar Abortusovis infections, New South Wales, Australia using MLST sequence data. Sequence types in the Enterobase database with five or fewer allele differences from the Australian ST768 (shaded in red) were identified and used to generate a phylogeny of related *Salmonella* isolates using maximum likelihood method. All branches with less than 50% bootstrap support were collapsed. The ST at each terminal branch is shown, as is the number of isolates in the Enterobase database assigned that ST and the number of allele differences from ST768. Available source and location data for *Salmonella *Abortusovis STs are displayed. Two letter abbreviations are used for country of origin. AU, Australia; D, allele difference from ST 768; DK, Denmark; FR, France; GB, Great Britain; H, human; IT, Italy; L, location; P, poultry; S, sheep; So., source; ST, sequence type.

Because the poultry-associated clade was distant from other *S.* *enterica* Abortusovis strains, we examined the relationship of that clade to other serovars by using MLST. We located 5 additional *Salmonella* Abortusovis isolates in Enterobase (*30*). Those isolates had STs assigned but lacked genomic data, including 1 ovine isolate from Denmark (ST730) in 1920 and 1 from Italy (ST202) in 1980, plus 1 avian isolate from Australia in 2009 (ST786). We found that isolates from outside Australia that had genomic data were assigned to ST373 (6 isolates) and ST8760 (1 isolate). The ST assigned to the 2009 isolate from Australia, ST768, was also assigned to all isolates in the poultry-associated clade in this study. We identified all STs that were similar to ST768 and generated a phylogeny from MLST allele sequences. That phylogeny demonstrated that the poultry-associated clade was more closely related to the ovine-associated *Salmonella* Abortusovis clade than to any other serovars (Figure 3).

### Estimation of MRCA for the Poultry-Associated Clade

*Salmonella* Abortusovis was observed in poultry in Australia beginning in 2009. We estimated the age of the poultry-associated clade by using temporal Bayesian analysis to determine whether that was the origin of the clade, or the clade was older. We estimated the date of the MRCA of the poultry-associated clade to be September 2004 (highest posterior density October 1998­–August 2009) with a mutation rate of 2.43 × 10^−7^ (highest posterior density 1.55 to 3.28 × 10^−7^) SNPs/site/year, equivalent to 1.19 (range 0.76–1.61) SNPs/genome/year.

### Plasmids in the Poultry-Associated Clade

To characterize the plasmid complement of the poultry-associated clade, we generated complete genomes, including closed plasmid sequences for 4 isolates: 180121-R1, 401964-R1, 405987-R1, and 405580-R1 (Table 2). We found that 405580-R1 carried no plasmids, but 180121-R1 and 401964-R1 contained the same 139-kb plasmid (pSAbAus), which carried the *Yersinia* high pathogenicity island (HPI) encoding yersiniabactin, a vertebrate lysozyme inhibitor (*ivy*), and a colicin secretion protein (*cvaA*). Isolate 405987-R1 carried a 60-kb plasmid that is identical to pSAbAus with a 79-kb deletion, which included the HPI, *ivy*, and *cvaA* loci. Isolate 401964-R1 also carried an additional 33-kb plasmid that is identical to pRHB32-C15_3 (GenBank accession no. CP057236.1) apart from 12 SNPs. Plasmid pRHB32-C15_3 was isolated from an *Escherichia coli* isolate on a sheep farm in the United Kingdom in 2017. We identified plasmid Inc types in all genomes and identified IncFIB(K)_1_Kpn3 in 45 genomes and on the 139-kb and 60-kb plasmids in complete genomes.

**Table 2:** Plasmids identified in a study of the emergence of poultry-associated human *Salmonella enterica* serovar Abortusovis infections, New South Wales, Australia*

Plasmid	Genomes		Length, bp	Virulence gene carriage	Inc type
	Complete	Draft†			
pSAbAus	3, 1 partial	39, 1 partial	139,222	Yersinia HPI, *ivy*, *cvaA*	IncFIB(K)_1_Kpn3
pRHB32-C15(3)	1	12	60,178	None	IncX1_1

*HPI, high-pathogenicity island. †Plasmid presence in draft genomes Inferred from reads.

We examined draft genomes of the remaining isolates for plasmids that we had identified in the complete genomes, and only detected pSAbAus in the poultry-associated clade. We found the complete plasmid in 42 isolates, including 2 complete genomes, and it was partially extant in 2 isolates, including 405987-R1, but was absent from 7 isolates, including 405580-R1 (Figure 2). We detected the pRHB32-C15_3 plasmid in 13 poultry-associated clade isolates (Appendix 2 Table 3).

Three genomes in the ovine-associated clade contained an IncFII(S)_1 gene and Spv toxin encoding genes *spvC*, *spvD*, and *spvR*, which are typical of *Salmonella* virulence plasmids, such as pSLT from *Salmonella* Typhimurium. Those 3 strains also contained regions 35,184­–38,729 bp in length that match to the pSLT plasmid at >97% identity. Taken together, those findings show that the 3 strains likely contain a *Salmonella* virulence plasmid. However, determining the makeup of that plasmid is difficult because no complete genomes were available in the ovine-associated clade. We did not observe any evidence of a classical virulence plasmid in the other 2 strains in the ovine-associated clade or the poultry-associated clade.

### Prophage Variation within the Poultry-Associated Clade

We identified prophage complements of poultry-associated clade isolates in the 4 complete genomes and identified 6 intact prophages and 1 questionable prophage (Table 3). We used the intact prophages to infer the prophage complement of isolates without complete genomes (Table 3; Appendix 2 Table 3). Of those isolates, Gifsy-2 was in all isolates from the poultry-associated clade and was the only prophage carrying a virulence factor, cytolethal distending toxin gene *cdtB* (Figure 2). All prophages were most similar to prophages in other *Salmonella* serovars, except Abortus_SfV, which was most similar to a prophage from *Shigella flexneri* (*31*).

**Table 3:** Prophages identified in a study of the emergence of poultry-associated human *Salmonella enterica* serovar Abortusovis infections, New South Wales, Australia*

Prophage†	Genomes		Length, kb	Condition§	Top PHASTEST hit	Virulence genes
	Complete	Draft‡				
Abortus_SEN8	4	43	57.2	Intact	Salmon_SEN8_NC_047753	None
Abortus_SfV	3	30	48.9	Intact	Entero_SfV_NC_003444	None
Abortus_Gifsy-2	4	43	30.1	Intact	Gifsy-2_NC_010393	*cdtB*
Abortus_Fels_1	4	43	33.2	Questionable	Salmon_Fels_1_NC_010391	None
Abortus_SPN9CC	3	18	39.1	Intact	Salmon_SPN9CC_NC_017985	None
Abortus_SPN3UB	1	22	46.7	Intact	Salmon_SPN3UB_NC_019545	None

*PHASTEST, PHAge Search Tool with Enhanced Sequence Translation (https://phastest.ca). †Prefixed with Abortus for *Salmonella* Abortusovis. ‡Inferred from reads. 4 poultry associated clade and 1 ovine associated clade isolates could not be examined as no reads were available. §Per PHASTEST.

### Pan-Genome Differences between Ovine- and Poultry-Associated Clades

To identify factors that might contribute to the different host range of the poultry- and ovine-associated clades, we examined pan-genomes to identify genes associated with either clade (Figure 2). There were 433 genes unique to the poultry-associated clade, 278 of which have no known function. Among the other 155 genes, 23 are found within prophages and 55 within the pSAbAus plasmid. The 77 chromosomal genes unique to the poultry-associated clade included 2 predicted fimbrial subunit genes, *cfaB* and *stfF*; an acid resistance chaperone protein gene, *hdeB*; and 10 (*eutEJGHABCLKR*) of the 17 (*eutSPQTDMNEJGHABCLKR*) genes of the ethanolamine utilization operon (Figure 2).

The ovine-associated clade contained 130 unique genes, 62 of which have no known function. Unique genes included *astA* and *sodC1*, which have previously been identified on a Gifsy-2 phage in this clade (*5*), as well as an outer membrane porin gene, *ompN*, and *hcp1*, a type 6 secretion system gene. However, because of the lack of a complete genome in the ovine-associated clade, we could not confirm the location of those specific genes.

All isolates contained intact or nearly intact chromosomal gene sets for *Salmonella* pathogenicity islands 1, 2, 3, and 24 (also called CS54) and for the enterobactin gene cluster and 3 types of fimbriae. We also detected virulence genes *ompA* and *mig-14* in all isolates.

### Ovine-Associated Clade Genomic Markers of Host Adaptation 

Genome size and pseudogene number can be indicators of host restriction. Therefore, we compared those metrics between the ovine- and poultry-associated clades. A representative ovine-associated clade genome was 315 Kbp (7.1%) smaller than a representative poultry-associated clade genome (4.43 Mbp vs. 4.74 Mbp). The average ovine-associated clade genome contained 446 (range 384–667) pseudogenes, but the poultry-associated clade had 237 (range 224­–248) pseudogenes (Appendix 1). That contrasting difference in the number of pseudogenes is similar in magnitude to generalist and host restricted *Salmonella* serovars. Generalist serovar Typhimurium has 201 and serovar Entertiditis has 320 pseudogenes; host-restricted serovar Typhi has 485 and serovar Gallinarium has 510 pseudogenes.

### Identification and Validation of *Salmonella* Abortusovis Serovar-Specific Gene Markers

We searched for candidate-specific gene markers for the poultry-associated clade and for *Salmonella *Abortusovis in the accessory genomes of a dataset of 106 common serovars including *Salmonella* Abortusovis (Appendix 1). We identified 2 *Salmonella* Abortusovis markers and 2 poultry-associated clade markers with 100% sensitivity and 100% specificity (Table 4) and provided DNA sequences of those 4 *Salmonella* Abortusovis serovar-specific gene markers (Appendix 2 Table 1).

**Table 4:** Sensitivity and specificity of serovar-specific gene markers in a study of the emergence of poultry-associated human *Salmonella enterica* serovar Abortusovis infections, New South Wales, Australia*

Specific genes	No. isolates	Length, bp	Sensitivity†	Specificity†		Protein function
				Identification, n = 2,314	Validation, n = 1,088	
Abortusovis-gene1	56	2,367	100	100	100	DUF6430
Abortusovis-gene2	56	330	100	100	100	Hypothetical
Abortusovis-AUS-gene1	51	831	100	100	100	DUF4238
Abortusovis-AUS-gene2	51	810	100	100	100	Hypothetical

*AUS, Australia; FN, false negative; TN, true negative; TP, true positive. †We used sensitivity and specificity as described in previous studies (*29*,*32*), where sensitivity is TP/(TP+FN) and specificity is TN/(TN+FP). Per those studies, we defined FN as the genomes from *Salmonella* Abortusovis lacking any of those same gene markers; FP as the genomes from other serovars containing all those same gene markers; TN as the genomes from other serovars lacking any of those same gene markers; and TP as the genomes from *Salmonella* Abortusovis containing all specific gene markers for *Salmonella* Abortusovis.

## Discussion

*Salmonella* Abortusovis is an ovine-adapted serovar and not known to cause disease in humans (*3*,*6*). The bacterium is endemic in several countries in Europe and Asia but has not been observed in the sheep population of Australia despite strong biosecurity surveillance and the serovar being notifiable (*8*–*10*,*33*). However, *Salmonella* Abortusovis was the second most frequent serovar in meat chickens in Australia in 2009 (*14*). We sequenced 47 isolates from poultry and human infections in Australia and compared those with 9 publicly available *Salmonella* Abortusovis genomes to understand the relationship between isolates from Australia and those from other countries and sources.

All isolates from Australia formed 1 closely related clade that is distant from the ovine-associated clade of *Salmonella* Abortusovis (Figures 1, 2). The 2004 MRCA date of the poultry-associated clade also indicates that it has existed in the NSW poultry flock for 15­–20 years, a finding supported by the reported observation of *Salmonella* Abortusovis in NSW in 2009 (*15*). The relationships of the isolates sampled suggest that the serovar in poultry in Australia originated from a single introduction, however because no related isolates have been identified, the source is unknown.

Analysis of the pan genomes of all *Salmonella* Abortusovis isolates revealed substantial differences between the poultry-associated (433 unique genes) and ovine-associated clades (130 unique genes). A subset of those unique genes can be attributed to the 5 prophages and 2 plasmids only found in the poultry-associated clade and 1 putative plasmid only found in the ovine-associated clade.

Although all *Salmonella* Abortusovis isolates shared a complement of virulence factors, we noted some key differences. The poultry-associated clade carried *Yersinia* HPI, *cvaA*, and *ivy* on a plasmid, and *cdtB* on a prophage. The *Yersinia* HPI is a virulence factor in multiple human pathogens (*34*–*37*), and *cdtB* is a virulence factor in both typhoidal and nontyphoidal *Salmonella* (*38*). Other poultry-associated clade–specific genes included *hdeB*, which is known to contribute to acid resistance in *Salmonella* Enteritidis (*39*), and *ivy*, which encodes a vertebrate lysozyme inhibitor that improves survival in human saliva (*40*). Both genes could improve the chances of survival of the bacterium through the human upper gastrointestinal tract. The colicin export protein gene, *cvaA*, and the ethanolamine utilization operon, *eut*, found in 10 of 17 genes of the poultry-associated clade might contribute to infection by enabling colonization through competition with gut microbiota (*41*). Of note, ethanolamine in the host also triggers the *eutR* gene to activate SPI2 expression and increases intramacrophage survival (*42*). The fimbrial gene *cfaB* is upregulated in *Salmonella* Typhi human infection and *stfF* is upregulated in *Salmonella* Typhimurium chicken infection, but their contributions to survival and virulence are unknown (*43*,*44*).

In the ovine-associated clade, the *sodC1* gene is essential for systemic disease in lambs (*5*). The Spv toxin was essential in a murine infection model (*6*,*7*). The *spv* genes and associated pSLT-like putative plasmid were only found in 3 of 5 ovine-associated clade isolates, suggesting that virulence within that clade might vary. Indeed, the severity of *Salmonella* Abortusovis infections in sheep is known to vary greatly (*45*). Virulence genes that are specific to the ovine-associated clade include *ompN*, which is associated with survival in macrophages in *Salmonella* Typhi (*46*), and *hcp*, which is required for killing of commensal bacteria in *Salmonella* Typhimurium (*47*). The clade-specific genes and gene sets described here might explain the potential differences in host range and disease type between the ovine-associated and poultry-associated clades.

*Salmonella* Abortusovis was previously thought to be host restricted in sheep (*45*). Host restriction often leads to adaptations, including a reduction in genome size and an increase in pseudogenization (*48*). The average number of pseudogenes in an ovine-associated clade isolate is double the average of a poultry-associated clade isolate, and the average genome was 7.1% smaller. The pseudogene count of the poultry-associated clade was also similar to those of generalist serovars, such as *Salmonella* Typhimurium and *Salmonella* Enteritidis, but the ovine-associated clade was similar to the host restricted serovars *Salmonella* Typhi and *Salmonella* Gallinarium. Those results suggest that the ovine-associated clade might be host adapted and the poultry-associated clade likely has a broader host range. That hypothesis is supported by the data in this study, namely, the ability of the poultry associated clade to infect both chickens and humans.

One of the plasmids in the poultry-associated clade is very closely related to an *E. coli* plasmid found on a sheep farm in the United Kingdom (*49*), suggesting that the poultry-associated clade was linked with sheep. However, the link is more likely to be indirect through the *E. coli* host.

The epidemiology of the isolates in this study demonstrated that the poultry-associated clade was widely distributed in NSW poultry and caused sporadic human infections. *Salmonella* Abortusovis was sampled across 8 years and was found in feed ingredients, in egg-laying hens, and in 20 different meat chicken farms from 2 regions. In addition, isolates from both region A and region B were found in each of the subclades in the phylogeny, suggesting that *Salmonella* Abortusovis has no geographic barriers. One exception was the egg layer hen population that formed a single group within subclade 2, indicating a single introduction. We also noted evidence for both long-term carriage of a single clone in 1 farm and multiple separate introductions into another farm. Isolation of *Salmonella* Abortusovis from feed ingredients suggests that transmission might have occurred through feed, but we found no identical isolates within short timespans in feed or chickens that would indicate direct transmission. Isolates from human infections were distributed across the phylogeny, suggesting that multiple separate transmission events to humans occurred; however, none caused large outbreaks. *Salmonella* Abortusovis does not efficiently transmit to humans from poultry (*50*), which might explain the low number of sporadic human cases despite the widespread detection in poultry.

The ability of the poultry-associated clade to colonize poultry and cause human disease, and the possibility that it might not cause systemic disease in lambs, make detection and differentiation of this clade from the ovine-associated clade useful. The genetic markers identified here will enable simple differentiation of the 2 *Salmonella* Abortusovis clades by using genomic data and potentially decrease root cause analysis time for detection of this novel *Salmonella* in the food industry. All markers described here could be used to produce PCR-based assays that would enable simple detection and differentiation of *Salmonella* Abortusovis clades, which is necessary because of their ability to either cause human infections or cause major losses of sheep flocks.

In conclusion, we examined the genomic epidemiology of *Salmonella* Abortusovis in poultry and human infections in NSW, Australia. The poultry-associated clade was only distantly related to existing examples of *Salmonella* Abortusovis and had key differences in virulence factors that suggest it might have differences in host range and disease type. Evidence suggests that the serotype has become endemic within the NSW poultry industry, where it can move between poultry facilities and to humans. Surveillance using the newly identified genetic markers will be vital for tracking transmission within poultry producing regions and to prevent any future outbreaks that could be caused by this serovar.

Appendix 1 Additional information on the emergence of poultry-associated human Salmonella enterica serovar Abortusovis infections, New South Wales, Australia.

Appendix 2 Isolate information and metadata used in a study of the emergence of poultry-associated human Salmonella enterica serovar Abortusovis infections, New South Wales, Australia.
