# Supplementary material for: Emergence of Poultry-Associated Human Salmonella enterica Serovar Abortusovis Infections, New South Wales, Australia
Source: Emerg Infect Dis. 2024 Apr;30(4):691–700. doi: 10.3201/eid3004.230958 (PMC10977856; doi:10.3201/eid3004.230958)
Supplement: Appendix 1 — Additional information on the emergence of poultry-associated human Salmonella enterica serovar Abortusovis infections, New South Wales, Australia. [file 23-0958-Techapp-s1.pdf]

Article DOI: https://doi.org/10.3201/eid3004.230958

EID cannot ensure accessibility for supplementary materials supplied by authors. Readers who have difficulty accessing supplementary content should contact the authors for assistance.

Emergence of Poultry-Associated Human *Salmonella enterica* Serovar Abortusovis Infections, New South Wales, Australia

Appendix 1

Genome Sequencing and Assembly Details

All isolates were sequenced using Illumina paired end sequencing with 150-bp read size using libraries produced by the Nextera DNA kit (Appendix 2 Table 1, https://wwwnc.cdc.gov/EID/article/30/4/23-0958-App2.xlsx). Assemblies from Illumina data were generated using shovill v1.1.0 (https://github.com/tseemann/shovill) with SPADES as assembler (*1*), using default settings. CheckM (v1.2.2) using command lineage_wf with default settings was used to evaluate contamination and completeness of the assemblies with all genomes having completeness over 98 and contamination less than 2 (Appendix 2 Table 1) (*2*). The serotype of all isolates were confirmed using SISTR version 1.1.1 with default settings (*3*). Four isolates were sequenced using Oxford Nanopore Gridion. Complete genomes were generated using micropipe with default settings (*4*) which performed hybrid assembly using nanopore reads and corresponding Illumina data for the four isolates.

Gene-Based Phylogeny of 7 STs Related to ST768

The phylogeny was generated by first querying Enterobase for all isolates within 5 alleles of ST768 which yielded 837 isolates (*5*). The metadata and allele profiles for these isolates were downloaded from the Enterobase *Salmonella* database along with allele sequences. Allele sequences for all loci were concatenated for each strain and used as an alignment, which was then used to generate a phylogeny using IQ-TREE v2.0.3 (default settings) (*6*).

Dataset and Temporal Signal for Bayesian Analysis

The dataset used in this analysis consisted of a tree generated by IQtree with default settings using core SNP alignments from 47 genomes sequenced in this study as they included month of isolation data (*6*). Temporal signal was estimated using Tempest (v1.5.3) with best fitting root selected by the heuristic residual mean squared method (*7*). A moderate temporal signal was detected (TempEst R^2 value of 0.303 and correlation coefficient of 0.55) allowing estimation of an MRCA age.

KMA Verification of Virulence Gene Detection

KMA results were filtered for 80% gene length and 80% identity as well as greater than 20% relative read depth. Relative read depth was calculated to normalize for sequencing depth of each isolate using the coverage of the target gene relative to the average depth of 7 housekeeping genes used in MLST (*aroC*, *dnaD*, *hemD*, *hisD*, *purE*, *sucA*, and *thrA*). Genes that were absent in Abricate output but had relative coverage of between 1% and 20% based on KMA were classed as inconclusive.

Identification and Validation of Specific *Salmonella* Serovar Abortusovis Gene Markers

The pan genome of a diverse set of serovars was examined to identify markers only present in Abortusovis in general and also the poultry associated clade specifically. A set of 2314 *Salmonella* isolates from 108 common *Salmonella* serovars including the 56 from this study were annotated using Prokka (v1.13.3) with default settings (*8*). Core and accessory genomes were then identified using roary v3.12.0 using an 80% sequence identity threshold (*9*). Separate genes specific to the poultry associated lineage and the Abortusovis serovar as a whole were identified using scoary (v1.6.16) with default settings (*10*). A further 1,088 *Salmonella* isolates from a diverse set of serovars that were not serovar Abortusovis were used to validate the specificity of the markers. Specificity of the markers to *Salmonella* was confirmed by searching against the NCBI nt/nr database using BLASTN (*11*).

Increase in Pseudogene Counts in Ovine-Associated Clade

The increase in pseudogene count predicted by pseudofinder in the ovine associated clade was due to a 4.8-fold increase in fragmented genes (171.6 vs 36.0 average), a 1.9 fold increase in truncated genes (152.0 vs 83.0 average) and a 2.5 fold increase in genes missing an ORF (34.6 vs 13.7 average) (*12*). Because the same pipeline with the same settings were used on all genomes which were assembled from the same type of data, the differences in pseudogene number are likely real rather than being an artifact of the analysis.

References

1. Bankevich A, Nurk S, Antipov D, Gurevich AA, Dvorkin M, Kulikov AS, et al. SPAdes: a new genome assembly algorithm and its applications to single-cell sequencing. J Comput Biol. 2012;19:455–77. PubMed https://doi.org/10.1089/cmb.2012.0021

2. Parks DH, Imelfort M, Skennerton CT, Hugenholtz P, Tyson GW. CheckM: assessing the quality of microbial genomes recovered from isolates, single cells, and metagenomes. Genome Res. 2015;25:1043–55. PubMed https://doi.org/10.1101/gr.186072.114

3. Yoshida CE, Kruczkiewicz P, Laing CR, Lingohr EJ, Gannon VP, Nash JH, et al. The Salmonella In Silico Typing Resource (SISTR): An Open Web-Accessible Tool for Rapidly Typing and Subtyping Draft Salmonella Genome Assemblies. PLoS One. 2016;11:e0147101. PubMed https://doi.org/10.1371/journal.pone.0147101

4. Murigneux V, Roberts LW, Forde BM, Phan MD, Nhu NTK, Irwin AD, et al. MicroPIPE: validating an end-to-end workflow for high-quality complete bacterial genome construction. BMC Genomics. 2021;22:474. PubMed https://doi.org/10.1186/s12864-021-07767-z

5. Zhou Z, Alikhan NF, Mohamed K, Fan Y, Achtman M; Agama Study Group. The EnteroBase user's guide, with case studies on *Salmonella* transmissions, *Yersinia pestis* phylogeny, and *Escherichia* core genomic diversity. Genome Res. 2020;30:138–52. PubMed https://doi.org/10.1101/gr.251678.119

6. Minh BQ, Schmidt HA, Chernomor O, Schrempf D, Woodhams MD, von Haeseler A, et al. IQ-TREE 2: New Models and Efficient Methods for Phylogenetic Inference in the Genomic Era. Mol Biol Evol. 2020;37:1530–4. PubMed https://doi.org/10.1093/molbev/msaa015

7. Rambaut A, Lam TT, Max Carvalho L, Pybus OG. Exploring the temporal structure of heterochronous sequences using TempEst (formerly Path-O-Gen). Virus Evol. 2016;2:vew007. PubMed https://doi.org/10.1093/ve/vew007

8. Seemann T. Prokka: rapid prokaryotic genome annotation. Bioinformatics. 2014;30:2068–9. PubMed https://doi.org/10.1093/bioinformatics/btu153

9. Page AJ, Cummins CA, Hunt M, Wong VK, Reuter S, Holden MT, et al. Roary: rapid large-scale prokaryote pan genome analysis. Bioinformatics. 2015;31:3691–3. PubMed https://doi.org/10.1093/bioinformatics/btv421

10. Brynildsrud O, Bohlin J, Scheffer L, Eldholm V. Rapid scoring of genes in microbial pan-genome-wide association studies with Scoary. Genome Biol. 2016;17:238. PubMed https://doi.org/10.1186/s13059-016-1108-8

11. Altschul SF, Gish W, Miller W, Myers EW, Lipman DJ. Basic local alignment search tool. J Mol Biol. 1990;215:403–10. PubMed https://doi.org/10.1016/S0022-2836(05)80360-2

12. Syberg-Olsen MJ, Garber AI, Keeling PJ, McCutcheon JP, Husnik F. Pseudofinder: Detection of Pseudogenes in Prokaryotic Genomes. Mol Biol Evol. 2022;39:msac153. PubMed https://doi.org/10.1093/molbev/msac153
